# Odor Fingerprinting of Chitosan and Source Identification of Commercial Chitosan: HS-GC-IMS, Multivariate Statistical Analysis, and Tracing Path Study

**DOI:** 10.3390/polym16131858

**Published:** 2024-06-28

**Authors:** Jin-Shuang Guo, Gang Lu, Fu-Lai Song, Ming-Yu Meng, Yu-Hao Song, Hao-Nan Ma, Xin-Rui Xie, Yi-Jia Zhu, Song He, Xue-Bo Li

**Affiliations:** 1 Characteristic Laboratory of Forensic Science in Universities of Shandong Province, Shandong University of Political Science and Law, Jinan 250014, China; 19861720030@163.com (M.-Y.M.); 13869930109@163.com (Y.-H.S.); marsgee_2005@outlook.com (H.-N.M.); 13953181539@163.com (X.-R.X.); zhu20051024@126.com (Y.-J.Z.); 15163209819@163.com (S.H.); lixuebo@sdupsl.edu.cn (X.-B.L.); 2 State Key Laboratory of Structural Chemistry, Fujian Institute of Research on the Structure of Matter, Chinese Academy of Sciences, Fuzhou 350002, China; 3 Key Laboratory of Colloid and Interface Chemistry of the Ministry of Education, School of Chemistry and Chemical Engineering, Shandong University, Jinan 250100, China; 4 Qingdao Health Ocean Biopharmaceutical Co., Ltd., Qingdao 266001, China; 13780624708@163.com

**Keywords:** chitosan, source identification, headspace-gas chromatography-ion mobility spectrometry (HS-GC-IMS), multivariate statistical analysis, odor fingerprinting

## Abstract

Chitosan samples were prepared from the shells of marine animals (crab and shrimp) and the cell walls of fungi (agaricus bisporus and aspergillus niger). Fourier-transform infrared spectroscopy (FT-IR) was used to detect their molecular structures, while headspace-gas chromatography–ion mobility spectrometry (HS-GC-IMS) was employed to analyze their odor composition. A total of 220 volatile organic compounds (VOCs), including esters, ketones, aldehydes, etc., were identified as the odor fingerprinting components of chitosan for the first time. A principal component analysis (PCA) revealed that chitosan could be effectively identified and classified based on its characteristic VOCs. The sum of the first three principal components explained 87% of the total variance in original information. An orthogonal partial least squares discrimination analysis (OPLS-DA) model was established for tracing and source identification purposes, demonstrating excellent performance with fitting indices R^2^X = 0.866, R^2^Y = 0.996, Q^2^ = 0.989 for independent variable fitting and model prediction accuracy, respectively. By utilizing OPLS-DA modeling along with a heatmap-based tracing path study, it was found that 29 VOCs significantly contributed to marine chitosan at a significance level of VIP > 1.00 (*p* < 0.05), whereas another set of 20 VOCs specifically associated with fungi chitosan exhibited notable contributions to its odor profile. These findings present a novel method for identifying commercial chitosan sources, which can be applied to ensure biological safety in practical applications.

## 1. Introduction

Chitosan is a component of deacetylated chitin, which widely exists in shrimp and crab shells, insect exoskeletons, and fungal cell walls [1,2]. Chitosan has cationic polyelectrolyte and multi-functional group reactivity and easy chemical modification [3], with the advantages of non-toxicity, non-irritation, excellent histocompatibility, and biodegradability, which have been intensely studied in industry, medicine, biology, food, and other fields [4,5,6,7,8,9,10,11,12]. For example, surgical sutures made of chitosan derivatives have the characteristics of being degradable, promoting healing, and not easily leaving scars [11,12]. Chitosan medical dressings have the functions of promoting wound healing, relieving pain, and anti-infection [9]. Cosmetics containing chitosan have favorable moisture absorption, conditioning, and antibacterial functions [1].

The majority of commercially available chitosan is prepared from marine animal crustaceans, such as shrimp shells and crab shells [13,14]. Chitin can be obtained after decalcification of shells of marine animal crustaceans and then deacetylation by strong alkali pyrolysis [7]. The process is mature, with a relatively high yield and low cost. Compared with marine chitosan extracted from shrimp and crab shells, fungal chitosan has the advantages of easily available raw materials, less pollution in the production process, and uniform physical and chemical properties of the obtained products [14,15,16]. However, research on fungal chitosan is still in the experimental phase, and production has not been industrialized due to the low yield of fungal chitosan and the immature process.

Chitosan obtained from shrimp and crab shells belongs to materials of animal origin. Because the application of animal tissues to human beings will increase the security risks of virus transmission, numerous countries have closely regulated the application and product control of biotechnology products from animals [17,18]. This means that fungal chitosan and its related products, being non-animal in origin, can enter the market easier and faster than shrimp and crab ones. Moreover, because fungal chitosan is scarce, costly, and lucrative, it is highly tempting for some unscrupulous merchants to replace fungal chitosan or dope it with inexpensive shrimp and crab chitosan. Therefore, it is of great practical significance to explore the detection and identification methods to identify the source of chitosan for the health and rights of consumers, to standardize market order, and to promote legislative management. However, thus far, there has been no report on a method of accurately identifying sources of chitosan.

Gas chromatography–ion mobility spectrometry (GC-IMS) combines the advantages of efficient separation of gas chromatography and rapid trace analysis of ion mobility spectrometry [19]. After secondary separation, three-dimensional spectra are obtained based on gas chromatography retention time, ion migration time, and signal intensity. In recent years, headspace-gas chromatography–ion mobility spectrometry (HS-GC-IMS) has been reported for tracing, classifying, identifying, or analyzing the state of samples by detecting volatile organic compounds (VOCs) [19,20,21]. As typical marine animals have a distinct seafood odor [22,23], it is expected that shrimp and crab chitosan may retain or maintain some volatile components from the shells of marine organisms.

Therefore, the objective of this work is to provide a method to identify sources of chitosan through the analysis of VOCs. The VOCs profile of chitosan was obtained using HS-GC-IMS and visually represented through odor fingerprinting. The identification of marine chitosan was established by comparing the characteristic VOCs profiles of chitosan derived from shrimp and crab shells with those obtained from fungal sources, such as agaricus bisporus and aspergillus niger. The proposed method was evaluated using multivariate statistical analysis techniques, including principal component analysis (PCA), orthogonal partial least squares discrimination analysis (OPLS-DA), and heatmap models. This research provides a reliable identification method for identifying, classifying, and tracing the source of chitosan circulating in the market to ensure biological safety in practical applications.

## 2. Materials and Methods

### 2.1. Chemicals and Reagents

The chemicals used in the chitosan synthesis were obtained from commercial sources and utilized without further purification. LC-MS-grade water and aspartic acid (purity ≥ 99.0%) for the HS-GC-IMS pretreatment of the samples were procured from Supelco Company (Darmstadt, Germany) and Weng Jiang Reagent (Shaoguan, China), respectively. The retention indices of the target analytes were calculated using a mixture of 2-butanone, 2-pentanone, 2-hexanone, 2-heptanone, 2-octanone, and 2-nonone purchased from Aladdin (Shanghai, China).

### 2.2. Chitosan Samples

**Figure 1:**
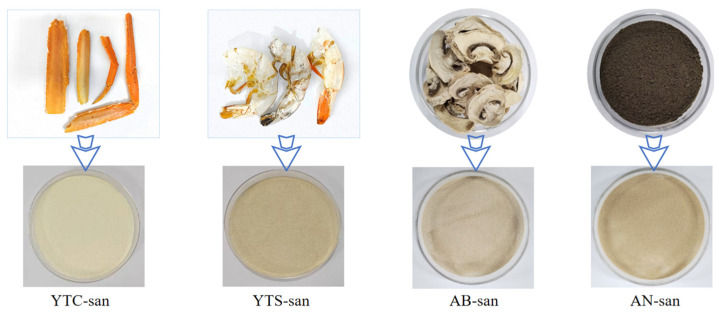
Raw material and the corresponding chitosan. Samples of crab chitosan (YTC-san), shrimp chitosan (YTS-san), agaricus bisporus umbrella chitosan (AB-san), and aspergillus niger mycelium chitosan (AN-san) were prepared from shells of crab legs, shells of shrimp, agaricus bisporus, and aspergillus niger, respectively.

The samples of crab chitosan (YTC-san), shrimp chitosan (YTS-san), agaricus bisporus umbrella chitosan (AB-san), and aspergillus niger mycelium chitosan (AN-san) were prepared in our laboratory to establish the method for analysis (Figure 1). The raw materials used, YTC-shell and YTS-shell, were retained and powdered for traceability analysis. Seven samples of commercial chitosan were obtained for validation testing, including CTS1, CTS2, CTS3, and CTS4, which were claimed to be shrimp and crab chitosan, as well as MF, FQ, and PBM, which were claimed to be fungal chitosan. All the relevant information regarding synthesis and purchase can be found in Text S1 and Table S1.

### 2.3. HS-GC-IMS Detection and Analysis

To investigate the odor characteristics of chitosan and its related samples in this study, an HS-GC-IMS analysis of the VOCs was conducted using a FlavourSpec^®^ Analyzer (G.A.S. Gesellschaft für analytische Sensorsysteme mbH, Dortmund, Germany) equipped with an automatic headspace sampler unit and a *β*-radiation source (Tritium (^3^H)) for ionization. The extraction of the VOCs from the sample was performed in situ within a headspace vial. In brief, 0.5 g of the test sample and 0.2 g of aspartic acid were accurately weighed and initially mixed in a 20 mL headspace glass sampling vial. Subsequently, 2 mL of H_2_O was added to moisten the powdery mixture. The samples were then sealed and incubated at 80 °C and 400 r·min^−1^ for 15 min. Afterward, a heated syringe installed at 85 °C was used to inject 0.5 mL of the incubated headspace gas for analysis purposes. The VOCs were isolated and analyzed using HS-GC-IMS. The chromatographic separation system employed a MAT-5 capillary column with dimensions of 15 m × 0.53 mm and a thickness of 1 μm. The column temperature was set at 60 °C. Nitrogen (purity ≥ 99.999%) served as the carrier gas, flowing at a preset rate of 2 mL·min^−1^ for the initial 2 min, gradually increasing to 10 mL·min^−1^ for the subsequent 8 min, further increasing to 100 mL·min^−1^ for the following 15 min, and maintaining this flow rate for the remaining 15 min. In the IMS detection system, the drift tube had a length of 98 mm. The linear voltage applied in the tube was 500 V·cm^−1^, and its temperature was maintained at 45 °C. Nitrogen (purity ≥ 99.999%) was used as drift gas with a flow rate of 150 mL·min^−1^. The ionization mode utilized was positive. Each sample required a detection time of approximately 30 min. Three test samples were injected and analyzed for each class.

### 2.4. Chemometrics

The chemometric analysis of the original HS-GC-IMS data was conducted using VOCal 0.4.03, an analysis software, along with its three plug-ins: Reporter, Gallery, and Dynamic Principal Component Analysis (PCA). Other internal instrumental software features, such as GC-IMS library search, laboratory analytical viewer (LAV), and CSV export, were essential for data analysis. Specifically, the NIST Kovats RI database in the GC-IMS library search provided a qualitative analysis of the VOCs. LAV was utilized for spectroscopic and semi-quantitative analysis. The CSV export function facilitated exporting the raw data, including the retention time, drift time, and peak volume information. Reporter and Gallery enabled a direct visual comparison of the different samples through three-dimensional (3D) spectra display, two-dimensional (2D) top-view plots presentation, and fingerprint comparisons. Dimension reduction techniques like PCA and orthogonal partial least-squares discriminate analysis (OPLS-DA) using SIMCA 18.0 were employed to uncover hidden tendencies in a sample cluster analysis.

## 3. Results and Discussion

### 3.1. Structure Analysis

**Figure 2:**
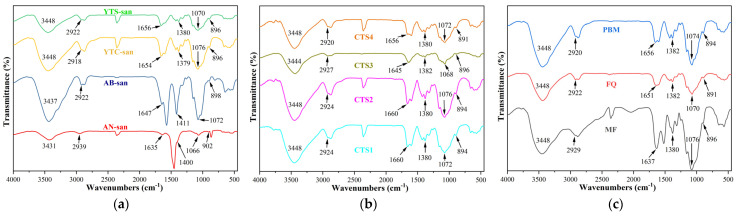
(**a**) FT-IR spectra of shrimp chitosan (YTS-san), crab chitosan (YTC-san), agaricus bisporus umbrella chitosan (AB-san), and aspergillus niger mycelium chitosan (AN-san) prepared in our laboratory for modeling purposes; (**b**,**c**) FT-IR spectra of CTS1–4, MF, FQ, and PBM obtained from the market for model validation.

Nowadays, various spectroscopic methods have been reported in the literature for confirming the structure of chitosan, including X-ray spectroscopy, Fourier-transform infrared spectroscopy (FT-IR), UV–Vis spectroscopy, mass spectrometry (MS), and so on [24,25]. Among these techniques, FT-IR spectroscopic analysis is widely considered the most established method for determining chitosan structure. In this study, both prepared chitosan (YTS-san, YTC-san, AB-san, and AN-san) and purchased chitosan (CTS1, CTS2, CTS3, CTS4, MF, FQ, and PBM) were investigated though FT-IR spectroscopic analysis. The obtained FT-IR spectra are presented in Figure 2.

The FT-IR spectra in Figure 2 were normalized to their maximum transmittance values and shifted on the same transmittance axis for comparison. The tested samples exhibited consistent infrared absorption from 500 cm^−1^ to 4000 cm^−1^, clearly indicating the absorption peaks of chitosan’s characteristic functional groups. A broad and intense band ranging from 3431 cm^−1^ to 3448 cm^−1^ was observed, suggesting the stretching vibration of O–H and N–H bonds [15,26]. Additionally, small peaks at approximately 2929 cm^−1^ were attributed to C–H stretching vibration [26,27]. Peaks around 1640 cm^−1^ and 1070 cm^−1^ corresponded to C=O stretching vibration related to amide I [15,26,28] and C–O–C bending vibration of the glucosidic bonds [26,28], respectively. Furthermore, symmetric and asymmetric bending of the –CH_2_– and –CH_3_ groups accounted for the peaks around 1380 cm^−1^. All these characteristic peaks mentioned above are in agreement with the Chinese pharmaceutical industry standard of Tissue engineering medical device products–Chitosan (YY/T 1699–2020). Moreover, the presence of peaks at about 898 cm^−1^ confirms the *β*-anomer conformation [15].

Seafood waste is considered an attractive source of chitosan [29], and crab and shrimp shells are widely utilized in the industry for chitosan production [14,30]. Commercial chitosan is a weakly cationic polysaccharide commonly obtained through partial deacetylation of chitin, primarily extracted from crab and shrimp shells [5,13,16]. However, the chemical, physicochemical, and biological properties of commercial chitosan remain largely unknown due to factors such as impurity content, average molecular weight, deacetylation degree, etc. [31]. FT-IR spectroscopy is a fundamental and reliable technique with numerous potential applications in biology and medicine owing to its non-interference nature and high sensitivity [32]. A significant amount of infrared spectral information of chitosan structure has been reported in the literature [15,26,27,28,32,33,34,35]. This information can serve as a reference for confirming the structure of chitosan. The consistency between the FT-IR results for all the tested samples and those reported in the literature indicates that there are no significant structural differences among different sources of chitosan. Therefore, it is necessary to develop new methods for tracing and identifying specific sources of chitosan. Moreover, the FT-IR results have ensured the structural reliability of the testing samples used in subsequent HS-GC-IMS detection for modeling and validation.

### 3.2. HS-GC-IMS Topographic Photographs

**Figure 3:**
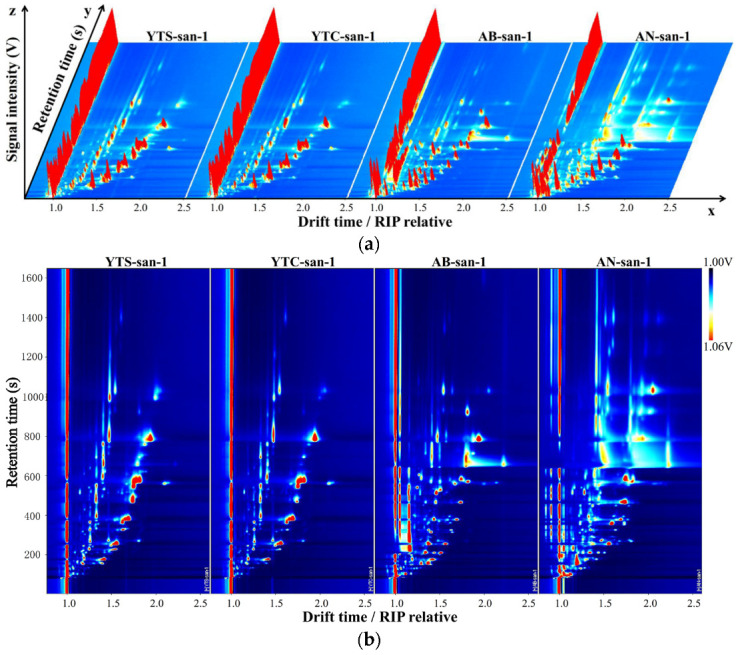
HS-GC-IMS topographic plots of shrimp chitosan (YTS-san), crab chitosan (YTC-san), agaricus bisporus umbrella chitosan (AB-san), and aspergillus niger mycelium chitosan (AN-san): (**a**) 3D topographic plots; (**b**) 2D topographic plots, as the top view of 3D topographic plots. The figure shows the results of one of three parallel tests for each sample.

The output plots in Figure 3 depict the typical results obtained from the HS-GC-IMS analysis of the prepared chitosan samples, namely, YTS-san, YTC-san, AB-san, and AN-san. The VOCs present in each sample were separated using a two-step process involving GC and IMS. Initially, the GC separated VOCs with different retention times, which were then soft-ionized by the ion source. Subsequently, based on their distinct ion masses and 1D collision cross-sections, these molecular ions experienced different drift velocities as they entered the linear drift electric field during periodic ion pulses at ambient pressure. Consequently, effective quadratic separation was achieved. In Figure 3a, raw three-dimensional topographic imaging of HS-GC-IMS is presented, wherein the drift time and retention time are plotted against the intensity of the ion signals. The drift time was normalized with respect to the reaction ion peak (RIP), denoted as RIP relative hereafter. The background color throughout this figure is blue, while the other colors indicate the signal strength of the VOCs: white represents low intensity, whereas red signifies high intensity. Notably, there existed a vertical signal wall at abscissa 1.0 corresponding to the RIP position, while each signal’s drift time was normalized relative to this position. Additionally, a two-dimensional plot (Figure 3b) was derived from the aforementioned three-dimensional topographic plot for better visualization purposes. It can be observed that major VOC signals associated with chitosan appear within a retention time range of 100 s to 1450 s and a normalized drift time range between 1.0 and 2.3 (relative to the RIP). Remarkably, more than 220 distinct peaks can be identified in Figure 3b, indicating the complex nature of the odor components present in chitosan.

The marine chitosan derived from shrimp and crab shells was visually distinguished by comparing 2D topographic plots, as illustrated in Figure S1. In each row, one sample of YTS-san or YTC-san was selected as the reference, while the remaining chitosan samples were subtracted from their respective references within the same row. The color brightness in the legend reflects the difference in spectral distribution of signal peaks. After deducting the reference, a broad white background appears for both YTS-san and YTC-san samples, indicating their similarity. However, when compared to YTS-san or YTC-san, significant differences of the VOCs were observed in fungal chitosan, AB-san and AN-san, through large red regions and blue plots in the subtracted spectra.

### 3.3. Analysis of VOCs in Chitosan

A qualitative analysis of the VOCs in chitosan from different sources is essential for investigating odor characteristics. According to the HS-GC-IMS topographic plots of the chitosan samples (Figure 3b), a total of 220 peaks were selected and presented in Table S2, comprising 178 qualitatively confirmed VOCs and 42 unidentified ones. Among the qualitatively confirmed VOCs, there were 18 pairs of monomers and dimers, resulting in a classification of 160 types of VOCs, including esters (32), ketones (26), aldehydes (25), alcohols (24), alkenes (9), and ethers (4), as well as other VOCs, such as heterocycles, carboxylic acids, and amines. The peak volume data were collected to perform a semi-quantitative analysis on the VOCs present in chitosan.

**Figure 4:**
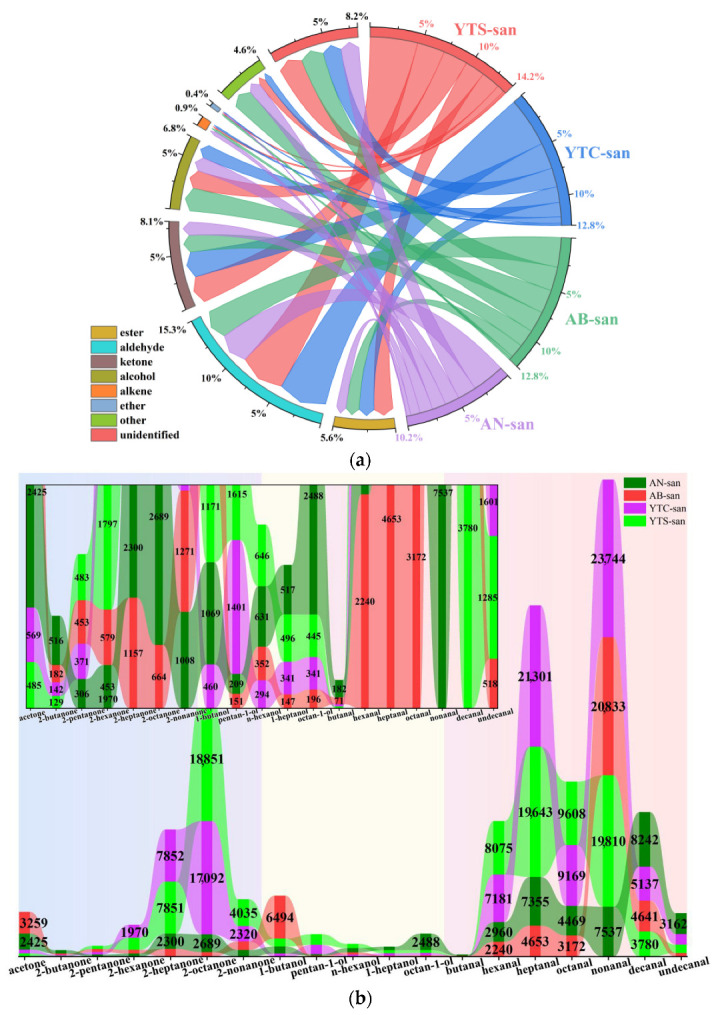
(**a**) Percentage composition of VOCs in different categories of chitosan; (**b**) contents of homologues in different chitosan samples with ketones, alcohols, and aldehydes listed from left to right. The inset image in (**b**) provides an enlarged view of the low content components. The results are reported as the intra-group mean of peak volumes obtained from HS-GC-IMS spectra.

The percentage composition of the VOC categories found in the different chitosan samples is illustrated in Figure 4a. Aldehydes were consistently identified as the primary component in each chitosan sample, followed by ketones or alcohols. Specifically, aldehydes accounted for nearly 5% of volatile compounds in YTS-san and YTC-san, and over 2.5% in AB-san and AN-san. Interestingly, Figure 4b reveals three series of homologues present in the complex VOC composition of chitosan: ketones ranging from C3 to C9, alcohols ranging from C4 to C8, and aldehydes ranging from C4 to C11 (excluding C5). The chitosan sample exhibited the highest overall levels of aldehyde homologues while having the lowest alcohol content. The positions of these homologues on HS-GC-IMS topographic plots (Figure S2) were determined based on their retention time on GC and drift time on IMS. Due to the regular variations in structural arrangements within each group of homologues, their signals are consistently positioned along two arc-like regions originating from the lower section of the 2D topographic plots of HS-GC-IMS and extending upwards.

In terms of the VOCs analysis, previous studies have focused on the utilization of chitosan and its derivatives as an anticorrosive material, with a primary emphasis on analyzing odors related to food spoilage rather than specifically examining the odor properties inherent to chitosan itself [36,37]. To our knowledge, this is the first report investigating the odor characteristics associated with chitosan.

### 3.4. VOCs Fingerprints and PCA Analysis

Compared to the non-targeted analysis method, selecting characteristic VOCs as markers can provide a purposeful strategy for species identification [38,39,40]. Therefore, the headspaces of the YTS-san, YTC-san, AB-san, and AN-san samples were sampled to collect the VOCs. These VOCs were then utilized to construct gallery plots that exhibit distinctive fingerprint features. Additionally, PCA was performed on the collected VOC data in order to visually represent the data and reveal hidden tendencies in the sample clustering. Specifically, all the samples of YTS-san, YTC-san, AB-san, and AN-san were prepared in our laboratory to ensure accurate identification of the raw materials’ species. The fingerprint features of the VOCs were explored using YTS-san and YTC-san extracted from shrimp and crab shells, respectively. Simultaneously, AB-san and AN-san, chosen as two fungal chitosan species, underwent testing and analysis using identical methods and parameters to determine if there were any differences in the VOC composition between marine chitosan and fungal chitosan.

**Figure 5:**
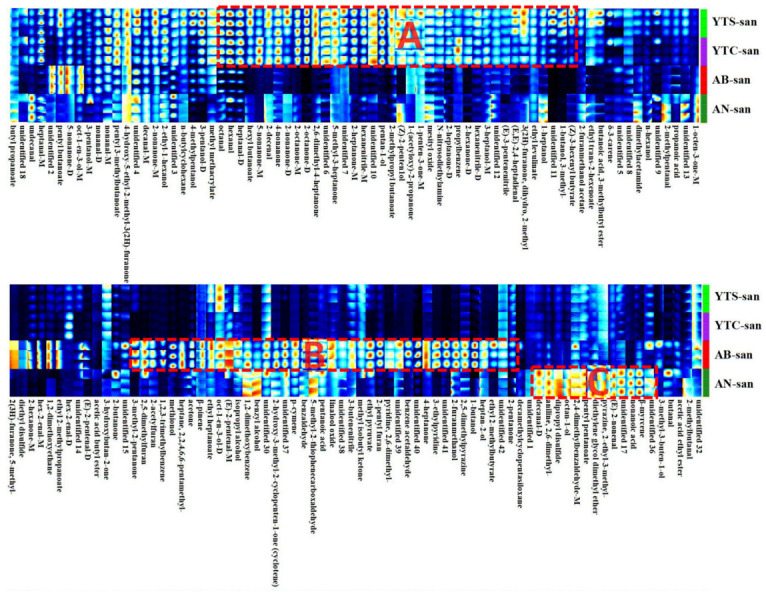
The main part of the gallery plot shows VOCs fingerprints of shrimp chitosan (YTS-san), crab chitosan (YTC-san), agaricus bisporus umbrella chitosan (AB-san), and aspergillus niger mycelium chitosan (AN-san). The intensity of the ion signal is described by the different colors. Regions A, B, and C represent characteristic VOCs of YTS-san and YTC-san, AB-san, and AN-san, respectively.

The gallery plots of Figure 5 and Figure S3e that each row represents the signal peaks of a specific flavor compound in different samples, while each column represents the signal peaks of various VOCs within one sample. The brighter spots indicate higher concentrations. Notably, the VOCs exhibited stability across the samples of each species, as evidenced by the consistent results obtained from the three parallel samples. The gallery plot visually presented both the similarities and differences in the VOC fingerprints among the YTS-san, YTC-san, AB-san, and AN-san. Remarkably similar total VOC profiles were observed for YTS-san and YTC-san, which can be attributed to Region A representing their characteristic VOC composition. Furthermore, Regions B and C revealed distinct increases in concentration for certain VOCs that contributed to additional distinguishing features between these samples. Additionally, low concentration components of some VOCs were also included in the gallery plot for comparison between different samples; these components might prove useful for detection and species identification.

**Figure 6:**
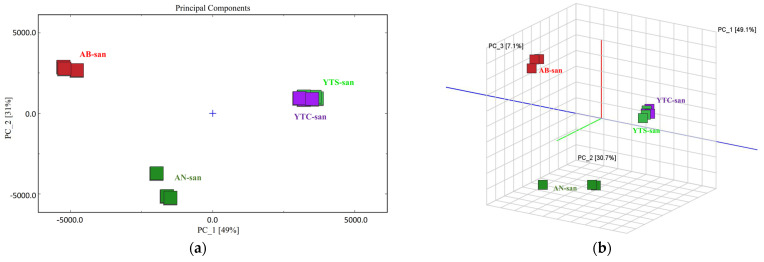
(**a**) PCA score plot of HS-GC-IMS spectra of different chitosan samples; (**b**) PCA 3D scatter plot of HS-GC-IMS spectra of different chitosan samples.

In terms of complex VOC components, it is valuable to investigate the concealed relationship between internal laws and system data in order to differentiate species or trace their sources [41,42]. PCA is an unsupervised learning method for dimensionality reduction that calculates principal component factors and is often employed as a preprocessing step before supervised modeling [43]. In this study, the PCA results were obtained based on all the selected characteristic peaks as variables. The PCA scores are presented in Figure 6a, revealing that the first two principal components determined by PCA accounted for 49% and 31% of the variance, respectively. The positions of YTS-san and YTC-san almost overlapped but were distinct from AB-san and AN-san. The 3D scatter plot (Figure 6b) also confirmed these clustering characteristics among inter-group differentiation, with the first three principal components accounting for a total of 87% (PC-1: 49.1%, PC-2: 30.7%, and PC-3: 7.1%) of the original information’s overall variance. The distances between the samples indicated differences in VOC composition. Although there were slight differences within the YTS-san and YTC-san groups, as evidenced by short sample distances, significant variations in the VOCs were observed between the regions when performing a PCA analysis on the chitosan samples, thus suggesting that shrimp and crab chitosans can be distinguished based on their VOC characteristics.

### 3.5. Establishment and Validation of a Model for Identification of Chitosan

The optimal model in supervised learning is obtained by training existing samples, which can then be used to map inputs to corresponding outputs for classification purposes [40]. This aligns with the objective of our study, which aims to establish and validate an identification model for chitosan sources. In this study, we employed OPLS-DA as a supervised learning method to further explore the differences between marine chitosan and fungal chitosan. A supervised OPLS-DA model was constructed using 220 peaks of VOCs detected by HS-GC-IMS as dependent variables and different origins as independent variables.

**Figure 7:**
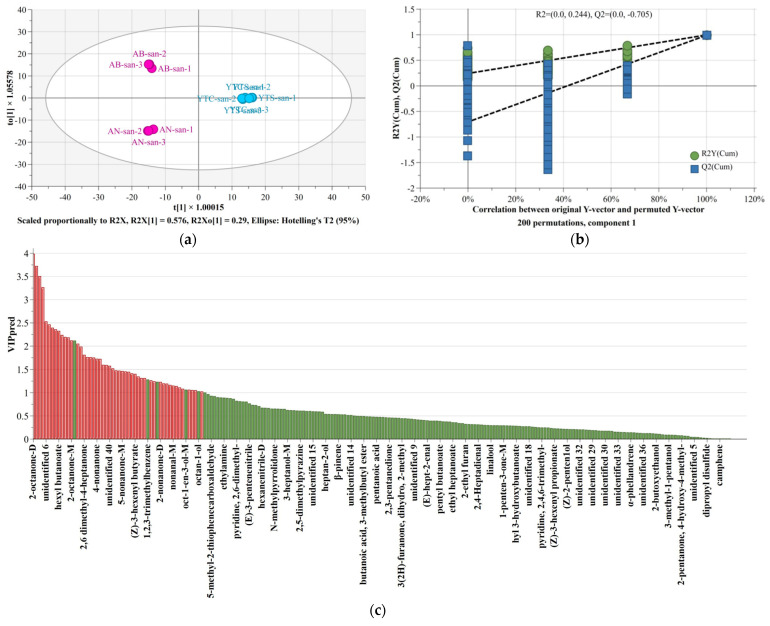
OPLS-DA model. (**a**) Score scatter plot; (**b**) permutations plot; (**c**) VIP values, in which red figures represent VOCs with calculation results of *p* < 0.05 and VIP > 1.00.

The score scatter plot of the OPLS-DA model in Figure 7a effectively distinguishes the chitosan samples extracted from two different raw materials, namely, marine animal shells and fungi. For clarity, Figure S4 presents a scatter plot of the default loading vectors for the first two components, while Figure S5 provides a magnified view at the center. In this model, the fitting index (R^2^X) for independent variables was 0.866, the fitting index (R^2^Y) for dependent variables was 0.996, and the prediction index (Q^2^) model performance was 0.989. It has been reported that fitting indices (R^2^) and prediction index (Q^2^) exceeding 0.5 indicate acceptable model results [44]. After conducting 200 displacement tests, as shown in Figure 7b, it can be observed that the regression line of Q^2^ passes through the abscissa with higher values on its right side compared to all the Q^2^ and R^2^ values on its left side. Moreover, the intersection point between the Q^2^ regression line and vertical axis is less than zero. These test results demonstrate that there is no overfitting in the OPLS-DA model and validate its efficiency accordingly. Therefore, it is believed that spectroscopic data obtained from HS-GC-IMS combined with this OPLS-DA model can be utilized for chitosan tracing purposes.

To further analyze the contribution rates of different VOCs in distinguishing marine chitosan derived from shrimp and crab shells and fungal chitosan derived from agaricus bisporus and aspergillus niger, we examined the VIP values for VOCs that differentiate between these two raw material regions using the OPLS-DA model. The variable importance of projection (VIP) included in the OPLS-DA model is presented in Figure 7c, illustrating the overall relevance of each variable in the model. The VIP values were utilized to evaluate the contribution of VOCs in the OPLS-DA model, with higher VIP values indicating a greater level of contribution. These results are displayed in a predicted VIP histogram arranged from highest to lowest value in Figure 7c. Detailed data on VIP and probability (*p*) can be found in Table S3. As the calculation results show, there were 49 distinct VOCs screened based on criteria such as *p* < 0.05 and VIP > 1.00 for chitosan originating from these two regions, including 12 ketones, 9 alcohols, 8 aldehydes, 4 esters, and 1 alkene, along with other categories comprising eight additional VOCs and seven unidentified compounds. These 49 highlighted VOCs were represented by red color both in the predictive VIP histogram and correlation scaled loadings scatter plot (Figure S6). Generally, variables with VIP values exceeding 1.00 were considered statistically significant [45]. As demonstrated by this correlation scaled load scatter plot analysis, each VOC’s contribution to different sample groups was clearly depicted through its position relative to each sample group symbol, as well as their respective distances apart from one another. The two groups of VOCs represented by red balls closely align with their corresponding blue group symbols, signifying a substantial contribution either from marine chitosan or fungal chitosan. It follows that the OPLS-DA model established in this study conclusively identifies the characteristic volatile organic compounds (VOCs) present in the complex odor components of chitosan, enabling their effective utilization for traceability and identification purposes. This model successfully addresses the technical limitation of FT-IR spectroscopic analysis, which solely confirms the structure of chitosan without providing information on its origin.

**Figure 8:**
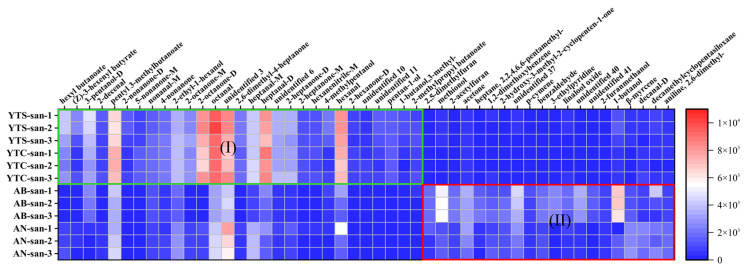
Heatmap of differential VOCs between chitosan samples derived from different raw materials.

The peak volumes of the 49 selected VOCs by the OPLS-DA model were input into a heatmap to further explore and identify the characteristic volatile substances of chitosan from different sources, as plotted in Figure 8. Region I contained 29 VOCs with significant levels found in shrimp/crab chitosan, such as hexyl butanoate, (Z)-3-hexenyl butyrate, and 3-pentanol. On the other hand, Region II consisted of 20 VOCs predominantly present in fungal chitosan, including 2,5-dimethylfuran, methionol, and 2-acetylfuran. In contrast to the fingerprints shown in Figure 5, there were a total of 38 VOCs in Region A and 55 VOCs collectively present in Regions B and C that subjectively represented characteristic VOCs of marine chitosan or fungal chitosan, respectively. The statistical analysis conducted using the OPLS-DA model demonstrated significant contributions from these identified VOCs for distinguishing between different sample groups. Comparing Figure 8 with Figure 5, 22 VOCs of Region I can be found in Region A, and 19 VOCs of Region II in Regions B and C. This observation indicates good agreement between the calculations performed by the OPLS-DA model and apparent patterns displayed by the fingerprints. Furthermore, seven additional VOCs of Region I, along with one more of Region II were located outside of Regions A, B, and C rather than being misclassified. These results validate that supervised OPLS-DA modeling can effectively explore relationships among large datasets to objectively identify differences among various chitosan samples.

The validation of the OPLS-DA model for chitosan identification was performed using the prediction function in the SIMCA 18.0 analysis software. The complete validation of the prediction model includes a work set, a test set, and a classification set. Table S4 shows all of the prediction results. The HS-GC-IMS data from the prepared modeling samples (YTS-san, YTC-san, AB-san, and AN-san) were appropriately utilized as the work set. The OPLS-DA model for chitosan identification automatically calculated the classification variable values (Y_Pred_) for the work set samples based on their assigned origins. Samples with 0.5 < Y_Pred_ ≤ 1.5 were classified as belonging to the producing area of the standard sample, while −0.5 ≤ Y_Pred_ ≤ 0.5 were not considered to originate from the standard sample’s origin. The test set comprised shrimp/crab chitosan samples obtained from different producers (CTS1, CTS2, CTS3, and CTS4). All of these samples met the requirement for classification as shrimp and crab chitosan (0.5 < Y_Pred_ (shrimp/crab chitosan) ≤ 1.5), thereby demonstrating that our model achieved an accuracy rate of 100% in predicting shrimp/crab chitosan. Subsequently, three batches of samples labeled as fungal chitosan (MF, FQ, and PBM) were purchased online and tested using HS-GC-IMS before being classified by the OPLS-DA model. The classification results reveal that only MF was categorized as fungal chitosan, with the Y_Pred_ values of 0.73, 0.74, and 0.82, whereas FQ and PBM were identified as shrimp/crab chitosan with their respective Y_Pred_ values falling between −0.5 and 0.5. On one hand, the research findings validate the identification capability of the chitosan traceability model; on the other hand, they also demonstrate that there is adulteration in the chitosan market and underscore the necessity of this traceability method for identifying commercial chitosan sources.

## 4. Conclusions

In conclusion, the odor fingerprinting of chitosan has been successfully established, revealing a consistent presence of series homologues of aldehydes, ketones, and alcohols. The VOCs data were collected from chitosan derived from crab and shrimp shells, as well as cell walls of agaricus bisporus and aspergillus niger using HS-GC-IMS. A total of 220 VOCs have been identified and input into a comprehensive method for tracing chitosan via multivariate chemometrics techniques, including PCA, OPLS-DA, and heatmap models. A PCA analysis revealed that chitosan could be effectively distinguished and classified based on its characteristic VOCs. The first three principal components accounted for 87% of the original information. Furthermore, an OPLS-DA model was established to trace the sources of chitosan with fitting indices R^2^X = 0.866, R^2^Y = 0.996, Q^2^ = 0.989 for independent variable fitting and model prediction accuracy, respectively. By employing OPLS-DA modeling along with a heatmap-based tracing path study, it was discovered that 49 VOCs significantly contributed to identifying the source of chitosan at a significance level of VIP > 1.00 and *p* < 0.05; among them were one set consisting of 29 VOCs notably contributing to marine chitosan and another set consisting of 20 VOCs contributing to fungi chitosan. The developed OPLS-DA model for source identification was successfully validated and applied to accurately identify the source of chitosan. This aforementioned method can be utilized to trace the origin of commercial chitosan products in order to detect adulterated derivatives in the market while ensuring accurate disclosure of biosafety information to consumers.

## Data Availability

Data are contained within the article.

## Supplementary Materials

The following supporting information can be downloaded at: https://www.mdpi.com/article/10.3390/polym16131858/s1. Text S1. Synthesis and samples. Text S2. Freeze-grinding process. Table S1. Detailed information on samples purchased. Table S2. HS-GC-IMS integration parameters of VOCs in chitosan. Figure S1. Comparison of 2D topographic plots. Figure S2. Locations of homologues in HS-GC-IMS topographic plots. Figure S3. Whole gallery plot of chitosan. Figures S4 and S5. Loading scatter plot of the OPLS-DA model. Table S3 and Figure S6. VOCs with high contributions in the OPLS-DA model. Table S4. Predicted results of the source identification model.
