# Supplementary material for: Odor Fingerprinting of Chitosan and Source Identification of Commercial Chitosan: HS-GC-IMS, Multivariate Statistical Analysis, and Tracing Path Study
Source: Polymers (Basel). 2024 Jun 28;16(13):1858. doi: 10.3390/polym16131858 (PMC11243783; doi:10.3390/polym16131858)
Supplement: Supplementary file 1 [file polymers-16-01858-s001.zip › polymers-3066058-supplementary.pdf]

polymers

Supplementary Materials: Odor Fingerprinting of Chitosan and Source Identification of Commercial Chitosan: HS-GC-IMS, Multivariate Statistical Analysis, and Tracing Path Study

Jin-Shuang Guo [1,2,*], Gang Lu [3,*], Fu-Lai Song [4], Ming-Yu Meng [1], Yu-Hao Song [1], Hao-Nan Ma [1], Xin-Rui Xie [1], Yi-Jia Zhu [1], Song He [1] and Xue-Bo Li [1]

[1] Characteristic Laboratory of Forensic Science in Universities of Shandong Province, Shandong University of Political Science and Law, Jinan, Shandong 250014, China
[2] State Key Laboratory of Structural Chemistry, Fujian Institute of Research on the Structure of Matter, Chinese Academy of Sciences, Fuzhou, Fujian 350002,China
[3] Key Laboratory of Colloid and Interface Chemistry of the Ministry of Education, School of Chemistry and Chemical Engineering, Shandong University, Jinan 250100, China
[4] Qingdao Health Ocean Biopharmaceutical Co., Ltd., Qingdao, Shandong 266001, China
* Correspondence: guojinshuang@sdupsl.edu.cn (J.-S.G.); ganglu@sdu.edu.cn (G.L.)

Table of Contents

1. Synthesis and samples (Text S1) 2
2. Freeze-grinding process (Text S2) 2
3. Detailed information on samples purchased (Table S1) 3
4. Comparison of 2D topographic plots (Figure S1) 3
5. HS-GC-IMS integration parameters of VOCs in chitosan (Table S2) 4
6. Locations of homologues in HS-GC-IMS topographic plots (Figure S2) 10
7. Whole gallery plot of chitosan (Figure S3) 11
8. Loading scatter plot of the OPLS-DA model (Figure S4, Figure S5) 12
9. VOCs with high contributions in the OPLS-DA model (Table S3, Figure S6) 13
10. Predicted results of the source identification model (Table S4) 14

1. Synthesis and samples (Text S1)

YTC-san and YTS-san: The samples of crab chitosan (YTC-san) and shrimp chitosan (YTS-san) were prepared under the same technological conditions and parameters. The synthetic process was divided into two parts, with the first part involving the preparation of chitin and the second part involving the preparation of chitosan. The crab and shrimp shells were used as raw materials without any crushing after cleaning. A mixture of hydrochloric acid (2.0 mol·L^{-1}) and crab/shrimp shells at a mass ratio of 40 : 1 was mechanically stirred for 3 hours at room temperature to achieve decalcification. The resulting reaction liquid was discarded, while the remaining materials were washed with H_2O until they were neutralized, followed by soaking them in a NaOH solution (2.5 mol·L^{-1}) for 2 hours to deproteinize them. After discarding the reaction liquid, washing them again until they were neutralized, and naturally drying them, crab chitin and shrimp chitin were obtained with yields of 13.04% and 14.86%, respectively. The next step involved preparing YTC-san and YTS-san by mixing crab/shrimp chitin with NaOH solution (12.5 mol·L^{-1}) at a mass ratio of 1 : 3, followed by an 8-hour reaction at 95 ℃. Lamellar forms of YTC-san and YTS-san were obtained after repeated washing with H_2O until neutralization, followed by natural drying, resulting in yields of 9.52% for YTC-san and 10.94% for YTS-san. To additionally analyze their structures, the lamellar samples of both YTC-san and YTS-san were ground into powder using a freeze grinder at -40 ℃. Details about this freeze-grinding method can be found in Text S2.

AB-san and AN-san: Agaricus bisporus umbrella chitosan (AB-san) and aspergillus niger mycelium chitosan (AN-san) were also prepared in our laboratory. The umbrellas of agaricus bisporus were selected and washed with H_2O. After drying them in an air-blower driver at 45 ℃ for 12 hours, the bisporus umbrellas were ground into powder. The powder was dispersed in a NaOH solution (1.0 mol·L^{-1}) at a mass ratio of 1 : 40 and stirred at 90 ℃ for 0.5 hours. The precipitate was then washed to a neutral pH and extracted at 95 ℃ for 6 hours using acetic acid (0.33 mol·L^{-1}) with a material-to-solution mass ratio of 1 : 100. Subsequently, the reaction system was adjusted to alkaline conditions at a pH of 10, and the supernatant was discarded. The obtained precipitate underwent deacetylation by adding NaOH, with a final concentration of 40% and a material-to-water mass ratio of 1 : 40. An equal amount of H_2O was added to the reaction system, followed by centrifugation to obtain the precipitate again. After washing the sediment to a neutral pH, it was heated at 100 ℃ for 2 hours, together with acetic acid, containing a three percent concentration. The resulting mixture was then centrifuged to obtain the supernatant, which was adjusted to weakly alkaline conditions with a pH range between 7 and 8. The insoluble substances obtained were collected through suction filtration and washed with ethanol before being vacuum-dried, resulting in an AB-san product yield reaching 4.50%. Similarly, employing identical process parameters as those for AB-san extraction, the AN-san product yield from aspergillus niger mycelium reached a 3.70% yield. The structures of both AB-san and AN-san were confirmed via their FT-IR spectra (Figure2a).

CTS1–4, MF, FQ, and PBM: Powdered samples of CTS1, CTS2, CTS3, and CTS4, ostensibly shrimp/crab chitosan, were purchased from four different manufacturers, and three different samples of MF, FQ, and PBM, ostensibly fungal chitosan, were purchased online. The product information is illustrated in Table S1, and the structures were confirmed by FT-IR spectra, as shown in Figures 2b and 2c.

2. Freeze-grinding process (Text S2)

Shrimp/crab shells, chitin, and chitosan can be pulverized through cryogenic grinding. A 0.2 g sample along with six steel balls (3.0 mm in diameter) are placed into a 2 mL PP freeze-grinding tube. The cover plate is tightly secured before placing the freeze-grinding tube into a grinder. The cryogenic grinding parameters are set at a frequency of 60 Hz, with precooling for 1 min and freezing and grinding at -40 ℃ for 2 min with an interval of

10 s, and the process is repeated eight times. Following this method of grinding, powdered samples with particle sizes smaller than 0.1 cm can be obtained.

3. Detailed information on samples purchased (Table S1)

Sample name	Batch number	Manufacturer / Distributor
CTS1	20220808	Qingdao Biotemed Biomaterials Co., Ltd. Qingdao, China
CTS2	20220918	Weifang Kehai Chitin Co., Ltd. Weifang, China
CTS3	20210730	Qingdao Honghai Biotechnology Co., Ltd. Qingdao, China
CTS4	20221213	Juxian Haibei Biotechnology Co., Ltd. Rizhao, China
MF	20230110	Online distributor, China
FQ	0220608	Online distributor, China
PBM	20220725	Online distributor, China

4. Comparison of 2D topographic plots (Figure S1)

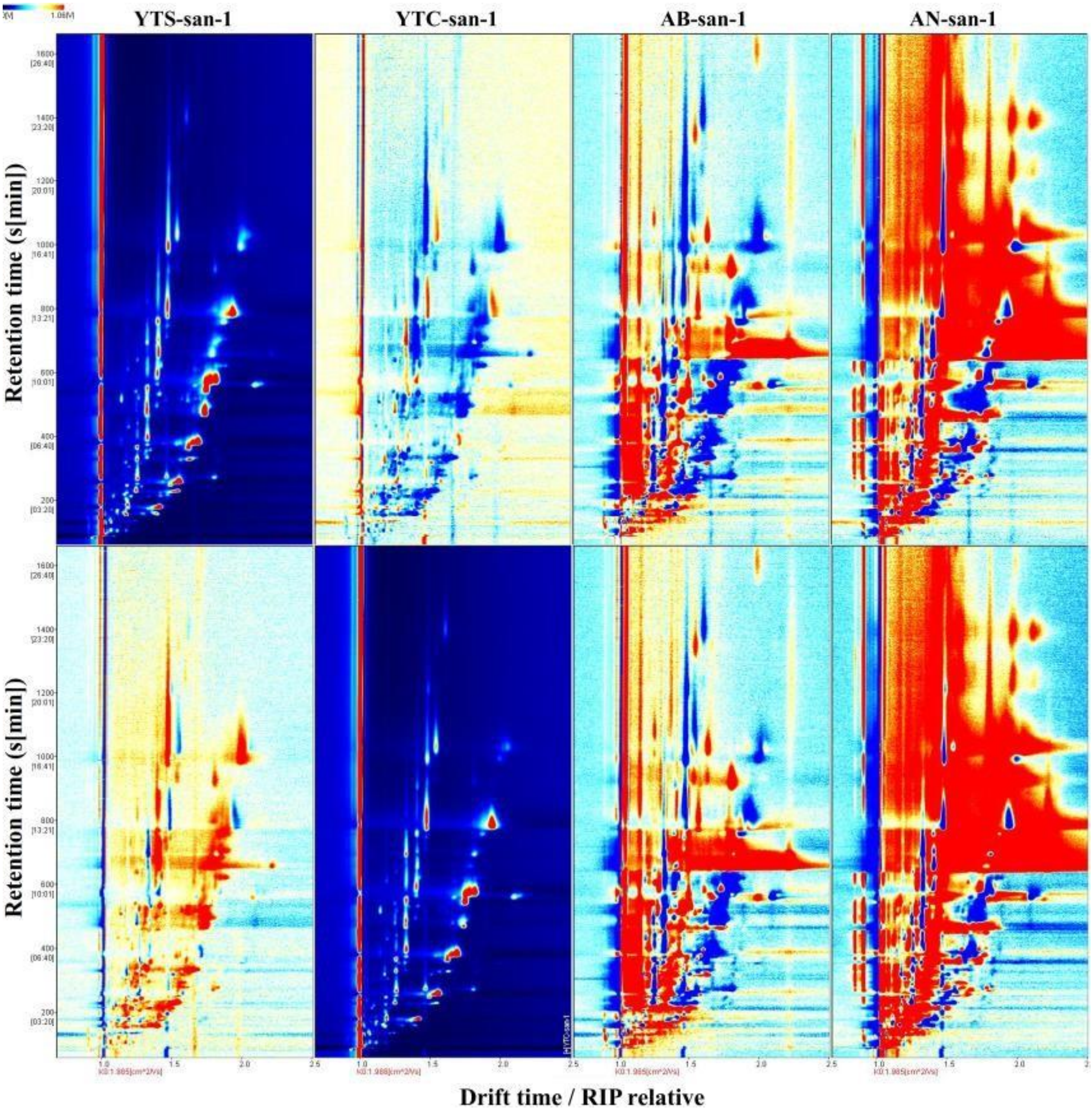

Figure S1. Comparison 2D topographic plots of shrimp chitosan (YTS-san), crab chitosan (YTC-san), agaricus bisporus umbrella chitosan (AB-san), and aspergillus niger mycelium chitosan (AN-san). YTS-san was selected as a reference in the first row, and YTC-san was selected as a reference in the next row.

5. HS-GC-IMS integration parameters of VOCs in chitosan (Table S2)

NO.	Compound name	CAS#	Formula	MW	RI	Rt [sec]	Dt [RIP relative]	Comment
1	undecanal	C112447	C11H22O	170.3	1282.5	1402.647	1.61344	
2	decanal	C112312	C10H20O	156.3	1188.6	1034	1.54865	Monomer
3	hexyl butanoate	C2639636	C10H20O2	172.3	1177.1	996.185	1.48339	
4	(Z)-3-hexenyl butyrate	C16491364	C10H18O2	170.3	1177.1	996.185	2	
5	decanal	C112312	C10H20O	156.3	1187.4	1030.213	2.05763	Dimer
6	decamethylcyclopentasiloxane	C541026	C10H30O5Si5	370.8	1154.4	925.399	1.811	
7	aniline, 2,6-dimethyl-	C87627	C8H11N	121.2	1158.2	936.792	1.55042	
8	1	unidentified	-	0	1178.5	1000.591	1.3829	
9	2-decenal	C3913711	C10H18O	154.3	1238.1	1214.625	1.48702	
10	(E)-2-nonenal	C18829566	C9H16O	140.2	1148.7	908.358	1.41214	
11	pentyl 3-methylbutanoate	C25415627	C10H20O2	172.3	1113.8	811.201	1.47849	
12	nonanal	C124196	C9H18O	142.2	1105	788.267	1.94365	Dimer
13	2-nonanone	C821556	C9H18O	142.2	1094.4	761.655	1.40919	Monomer
14	2-nonanone	C821556	C9H18O	142.2	1093.3	758.943	1.87918	Dimer
15	octan-1-ol	C111875	C8H18O	130.2	1075.3	715.849	1.46985	
16	5-nonanone	C502567	C9H18O	142.2	1066.8	696.2	1.33455	Monomer
17	pentyl butanoate	C540181	C9H18O2	158.2	1070.4	704.396	1.41015	
18	5-nonanone	C502567	C9H18O	142.2	1066.4	695.386	1.81725	Dimer
19	2	unidentified	-	0	1050.7	660.884	1.79661	
20	dipropyl disulfide	C629196	C6H14S2	150.3	1088.8	747.763	1.47273	
21	nonanal	C124196	C9H18O	142.2	1100.9	777.859	1.477	Monomer
22	4-nonanone	C4485090	C9H18O	142.2	1029	615.798	1.33649	
23	2-ethyl-1-hexanol	C104767	C8H18O	130.2	1020.1	598.188	1.40775	
24	2-octanone	C111137	C8H16O	128.2	995	551.065	1.33447	Monomer
25	oct-1-en-3-ol	C3391864	C8H16O	128.2	979.1	518.8	1.15034	Monomer
26	2-furanmethanol acetate	C623176	C7H8O3	140.1	986.7	533.984	1.41921	
27	2-octanone	C111137	C8H16O	128.2	993.6	548.029	1.75163	Dimer
28	octanal	C124130	C8H16O	128.2	1011.1	581.053	1.81844	
29	δ-3-carene	C13466789	C10H16	136.2	1000.7	561.694	2.1036	
30	3	unidentified	-	0	1009.9	578.739	1.75601	
31	2-pentyl furan	C3777693	C9H14O	138.2	990.2	541.012	1.25113	
32	1-octen-3-one	C4312996	C8H14O	126.2	980	520.549	1.27241	Monomer
33	1-heptanol	C111706	C7H16O	116.2	967.9	497.239	1.40068	
34	(E,E)-2,4-heptadienal	C4313035	C7H10O	110.2	977.1	514.893	1.62101	
35	4	unidentified	-	0	953.4	470.785	1.74049	
36	2,6-dimethyl-4-heptanone	C108838	C9H18O	142.2	961.3	484.976	1.32889	
37	ethyl trans-2-hexenoate	C27829727	C8H14O2	142.2	1032	621.916	1.81996	
38	n-butylcyclohexane	C1678939	C10H20	140.3	1042.1	624.54	1.26141	
39	5	unidentified	-	0	1004.2	568.089	2.14309	
40	butanoic acid, 3-methylbutyl ester	C109193	C9H18O2	158.2	1032.2	622.221	1.88828	
41	heptanal	C111717	C7H14O	114.2	909.5	398.703	1.33345	Monomer

42	heptanal	C111717	C7H14O	114.2	901.2	386.319	1.69505	Dimer
43	6	unidentified	-	0	899.1	383.39	1.65463	
44	2-heptanone	C110430	C7H14O	114.2	888.5	368.397	1.62538	Dimer
45	5-methyl-3-heptanone	C541855	C8H16O	128.2	936.5	441.607	1.26766	
46	7	unidentified	-	0	929.4	429.954	1.19809	
47	2-heptanone	C110430	C7H14O	114.2	888.4	368.205	1.25982	Monomer
48	hexanenitrile	C628739	C6H11N	97.2	856.9	327.881	1.25982	Monomer
49	4-methylpentanol	C626891	C6H14O	102.2	857.3	328.301	1.63003	
50	N-nitrosodiethylamine	C55185	C4H10N2O	102.1	893.1	374.771	1.54453	
51	8	unidentified	-	0	896.4	379.419	1.5668	
52	hexanenitrile	C628739	C6H11N	97.2	856.5	327.316	1.56881	Dimer
53	dimethylacetamide	C127195	C4H9NO	87.1	880.2	357.253	1.35273	
54	n-hexanol	C111273	C6H14O	102.2	862.8	335.008	1.33026	
55	9	unidentified	-	0	806.2	271.933	1.81069	
56	2-hexanone	C591786	C6H12O	100.2	781.1	247.793	1.18897	Monomer
57	mesityl oxide	C141797	C6H10O	98.1	790.3	256.405	1.44133	
58	hexanal	C66251	C6H12O	100.2	793.4	259.377	1.55877	
59	2-hexanone	C591786	C6H12O	100.2	781.4	248.097	1.49538	Dimer
60	10	unidentified	-	0	789	255.208	1.52309	
61	11	unidentified	-	0	759.8	228.508	1.54691	
62	pentan-1-ol	C71410	C5H12O	88.1	760.7	229.259	1.25499	
63	2-methylpentanal	C123159	C6H12O	100.2	740.9	212.558	1.21699	
64	hex-2-enal	C505577	C6H10O	98.1	849.7	319.242	1.17981	Monomer
65	1-(acetyloxy)-2-propanone	C592201	C5H8O3	116.1	837.8	305.592	1.20619	
66	3-heptanol	C589822	C7H16O	116.2	878.2	354.657	1.32366	Monomer
67	12	unidentified	-	0	915.3	407.577	1.2609	
68	ethyl 2-methylpropanoate	C97621	C6H12O2	116.2	755.4	224.649	1.18979	
69	3-methyl-2-pentanone	C565617	C6H12O	100.2	748	218.43	1.16993	
70	propanoic acid	C79094	C3H6O2	74.1	742.3	213.698	1.09077	
71	13	unidentified	-	0	776.5	243.516	1.29323	
72	3(2H)-furanone, dihydro, 2-methyl	C3188009	C5H8O2	100.1	754.5	223.89	1.42815	
73	14	unidentified	-	0	743.2	214.49	1.4248	
74	1-butanol, 3-methyl-	C123513	C5H12O	88.1	759.6	228.332	1.50741	
75	2-methylpropyl butanoate	C539902	C8H16O2	144.2	954.7	473.065	1.32463	
76	(E)-2-pentenal	C1576870	C5H8O	84.1	753.4	223.008	1.10358	Monomer
77	(E)-2-pentenal	C1576870	C5H8O	84.1	753.2	222.783	1.355	Dimer
78	2,5-dimethylfuran	C625865	C6H8O	96.1	741.9	213.429	1.34281	
79	acetic acid butyl ester	C123864	C6H12O2	116.2	803.6	269.372	1.61373	
80	hex-2-enal	C505577	C6H10O	98.1	849.1	318.517	1.50991	Dimer
81	(Z)-2-penten1ol	C1576950	C5H10O	86.1	759	227.756	1.45825	
82	3-(methylsulfanyl)propanol (methionol)	C505102	C4H10OS	106.2	979.7	519.987	1.46524	
83	2-acetylfuran	C1192627	C6H6O2	110.1	896.9	380.203	1.44741	
84	3-pentanol	C584021	C5H12O	88.1	695.6	178.884	1.1947	Monomer
85	acetone	C67641	C3H6O	58.1	478.1	103.179	1.11958	
86	3-pentanol	C584021	C5H12O	88.1	695.8	179.03	1.42005	Dimer
87	methyl methacrylate	C80626	C5H8O2	100.1	693	177.097	1.38864	
88	(E)-3-Pentenenitrile	C16529661	C5H7N	81.1	690.6	175.515	1.3673	

89	3-hydroxybutan-2-one	C513860	C4H8O2	88.1	687.2	173.56	1.33714	
90	2-butanone	C78933	C4H8O	72.1	584.7	134.514	1.24432	
91	1,2-dimethoxyethane	C110714	C4H10O2	90.1	650.4	158.385	1.30566	
92	1-penten-3-one	C1629589	C5H8O	84.1	680.7	170.798	1.07902	Monomer
93	2,4-dimethylbenzaldehyde	C15764166	C9H10O	134.2	1202.2	1080.644	1.26333	Monomer
94	pentyl pentanoate	C2173560	C10H20O2	172.3	1154.2	924.846	1.48375	
95	triacetin	C102761	C9H14O6	218.2	1325.1	1611.182	1.99343	
96	propanedioic acid, diethyl ester	C105533	C7H12O4	160.2	1072.1	708.216	1.258	
97	1,2,3-trimethylbenzene	C526738	C9H12	120.2	1016.6	591.511	1.15691	
98	α-phellandrene	C99832	C10H16	136.2	1002.7	565.504	1.22257	
99	ethyl heptanoate	C106309	C9H18O2	158.2	1082.8	733.414	1.4084	
100	15	unidentified	-	0	1001.5	563.276	2.01403	
101	octamethylcyclotetrasiloxane	C556672	C8H24O4Si4	296.6	991.2	543.093	1.68095	
102	heptane, 2,2,4,6,6-pentamethyl-	C13475826	C12H26	170.3	995.6	552.245	1.15795	
103	trimethylpyrazine	C14667551	C7H10N2	122.2	994.4	549.729	1.62515	
104	2,4-Heptadienal	C5910850	C7H10O	110.2	985.5	531.464	1.61473	
105	pyrazine, 2-ethyl-3-methyl-	C15707230	C7H10N2	122.2	995.1	551.154	1.59326	
106	diethylene glycol dimethyl ether	C111966	C6H14O3	134.2	955.9	475.217	1.6213	
107	β-pinene	C127913	C10H16	136.2	966.6	494.857	1.21093	
108	pyridine, 2,4,6-trimethyl-	C108758	C8H11N	121.2	986.1	532.732	1.58887	
109	camphene	C79925	C10H16	136.2	937.5	443.301	1.71394	
110	dimethyl trisulfide	C3658808	C2H6S3	126.3	939.3	446.253	1.304	
111	2(3H)-furanone, dihydro-5-methyl-	C108292	C5H8O2	100.1	932.4	434.855	1.12245	
112	butyl acrylate	C141322	C7H12O2	128.2	873.7	348.846	1.70136	
113	3-heptanol	C589822	C7H16O	116.2	876.4	352.291	1.6513	Dimer
114	16	unidentified	-	0	728.2	202.571	1.45283	
115	butyl formate	C592847	C5H10O2	102.1	728.6	202.895	1.5051	
116	17	unidentified	-	0	850.2	319.881	1.29771	
117	4-heptanone	C123193	C7H14O	114.2	870.2	344.276	1.22526	
118	butyl propanoate	C590012	C7H14O2	130.2	906.8	394.702	1.28453	
119	18	unidentified	-	0	805.7	271.456	1.75913	
120	2(3H)-furanone, 5-methyl-	C591128	C5H6O2	98.1	871	345.405	1.11204	
121	diethyl disulfide	C110816	C4H10S2	122.2	915.2	407.412	1.13359	
122	19	unidentified	-	0	820.5	286.627	1.26306	
123	ethyl trans-2-butenoate	C623701	C6H10O2	114.1	816.6	282.611	1.55693	
124	20	unidentified	-	0	812.3	278.091	1.60014	
125	21	unidentified	-	0	810.7	276.502	1.64037	
126	2-butanol	C78922	C4H10O	74.1	604.6	141.336	1.31868	Dimer
127	2-butanol	C78922	C4H10O	74.1	603.5	140.947	1.14826	Monomer
128	N,N-diethylethanamine	C121448	C6H15N	101.2	705.4	185.719	1.46737	
129	2-ethyl furan	C3208160	C6H8O	96.1	703.5	184.348	1.29897	
130	methylpyrazine	C109080	C5H6N2	94.1	824	290.347	1.06818	
131	22	unidentified	-	0	707.8	187.373	1.23229	
132	oct-1-en-3-ol	C3391864	C8H16O	128.2	979.3	519.28	1.58345	Dimer
133	2-n-butylfuran	C4466244	C8H12O	124.2	884.9	363.56	1.17626	

134	cumin aldehyde	C122032	C10H12O	148.2	1194.3	1053.412	1.87876	
135	2,4-dimethylbenzaldehyde	C15764166	C9H10O	134.2	1202.8	1082.761	1.74319	Dimer
136	anisaldehyde	C123115	C8H8O2	136.1	1226.3	1168.645	1.66187	
137	linalool	C78706	C10H18O	154.3	1109.2	798.987	1.7048	
138	cis-rose oxide	C3033236	C10H18O	154.3	1108.6	797.454	1.81781	
139	4-hydroxy-5-ethyl-2-methyl-3(2H)-fur anone	C27538096	C7H10O3	142.2	1119.6	826.388	1.32916	
140	23	unidentified	-	0	1090.8	752.709	1.32748	
141	α-terpinolene	C586629	C10H16	136.2	1084.9	738.347	1.30035	
142	(Z)-3-hexenyl propionate	C33467742	C9H16O2	156.2	1070.1	703.807	1.89148	
143	1-octen-3-one	C4312996	C8H14O	126.2	981.6	523.799	1.68266	Dimer
144	(E)-hept-2-enal	C18829555	C7H12O	112.2	960.5	483.587	1.66116	
145	2,6-dimethylpyrazine	C108509	C6H8N2	108.1	916.2	408.965	1.55231	
146	24	unidentified	-	0	927.8	427.239	1.5675	
147	25	unidentified	-	0	915.6	407.992	1.96668	
148	26	unidentified	-	0	970.5	502.143	1.25467	
149	propylbenzene	C103651	C9H12	120.2	958.1	479.164	1.25494	149
150	ethyl levulinate	C539888	C7H12O3	144.2	1026.2	610.35	1.19617	150
151	N-methylpyrrolidone	C872504	C5H9NO	99.1	1032.3	622.492	1.42923	151
152	5-ethyldihydro-2(3H)-furanone	C695067	C6H10O2	114.1	1035.1	628.186	1.17813	152
153	β-ocimene	C13877913	C10H16	136.2	1040.7	639.75	1.6814	153
154	1,8-cineol	C470826	C10H18O	154.3	1040.4	639.025	1.73873	154
155	3-octen-1-ol, (Z)-	C20125842	C8H16O	128.2	1046.3	651.374	1.73528	155
156	27	unidentified	-	0	1030.8	619.425	1.74724	156
157	2-methylpentanoic acid	C97610	C6H12O2	116.2	1025.8	609.476	1.57392	157
158	(Z)-3-hexenyl acetate	C3681718	C8H14O2	142.2	989.3	539.184	1.82473	158
159	5-methylfurfural	C620020	C6H6O2	110.1	940	447.776	1.12443	159
160	thiolan-3-one	C1003049	C4H6OS	102.2	921.7	417.515	1.19472	160
161	cyclohexanone	C108941	C6H10O	98.1	881.1	358.445	1.14649	161
162	isovaleric acid	C503742	C5H10O2	102.1	877.2	353.282	1.21898	162
163	2-butoxyethanol	C111762	C6H14O2	118.2	889.5	369.703	1.19894	163
164	28	unidentified	-	0	912.3	402.989	1.19897	164
165	29	unidentified	-	0	946.6	458.827	1.2596	165
166	α-pinene	C80568	C10H16	136.2	937.2	442.737	1.67035	166
167	ethyl acetoacetate	C141979	C6H10O3	130.1	927.8	427.307	1.60278	167
168	ethyl 3-hydroxybutanoate	C5405414	C6H12O3	132.2	949.4	463.644	1.64658	168
169	30	unidentified	-	0	907.6	395.793	1.61259	169
170	31	unidentified	-	0	922.2	418.401	1.68543	170
171	32	unidentified	-	0	863.9	336.465	1.48347	171
172	33	unidentified	-	0	808.6	274.368	1.84782	172
173	cyclopentanone	C120923	C5H8O	84.1	780.3	247.068	1.10405	173
174	3-methyl-2-butenal	C107868	C5H8O	84.1	790.8	256.951	1.08536	174
175	2-methyl-2-pentenal	C623369	C6H10O	98.1	825.6	292.138	1.49505	175
176	34	unidentified	-	0	837.9	305.708	1.5006	176
177	2-pentanone, 4-hydroxy-4-me-thyl-	C123422	C6H12O2	116.2	837.6	305.377	1.53117	177
178	3-methyl-1-pentanol	C589355	C6H14O	102.2	828.9	295.691	1.62007	178
179	(E)-2-hexenal	C6728263	C6H10O	98.1	823.4	289.736	1.5288	179

180	2-methylbutanoic acid	C116530	C5H10O2	102.1	830.1	297.045	1.20693	
181	35	unidentified	-	0	815.9	281.852	1.28511	
182	pyridine, 2,6-dimethyl-	C108485	C7H9N	107.2	868.4	342.033	1.08284	
183	2-pentanone	C107879	C5H10O	86.1	670.1	166.335	1.11778	
184	isopropyl alcohol	C67630	C3H8O	60.1	499.8	108.895	1.23452	
185	ethylamine	C75047	C2H7N	45.1	412	87.545	1.0956	
186	2,3-pentanedione	C600146	C5H8O2	100.1	658.7	161.68	1.28365	
187	1-octene	C111660	C8H16	112.2	787.5	253.802	1.46084	
188	36	unidentified	-	0	580.1	132.97	1.21618	
189	1-penten-3-one	C1629589	C5H8O	84.1	687.3	173.616	1.30727	Dimer
190	butanal	C123728	C4H8O	72.1	587.4	135.431	1.28734	
191	N-nitrosodimethylamine	C62759	C2H6N2O	74.1	740.8	212.5	1.21656	
192	Nonanoic acid	C112050	C9H18O2	158.2	1241.5	1227.881	1.56277	
193	1,2-dimethoxybenzene	C91167	C8H10O2	138.2	1112.1	806.63	1.57682	
194	benzyl alcohol	C100516	C7H8O	108.1	1043.3	645.01	1.50443	
195	2-hydroxy-3-methyl-2-cyclo-penten-1-o ne (cyclotene)	C80717	C6H8O2	112.1	1006.2	571.835	1.50452	
196	37	unidentified	-	0	1049.5	658.283	2.22716	
197	benzene acetaldehyde	C122781	C8H8O	120.2	1024	605.982	1.2686	
198	p-cymene	C99876	C10H14	134.2	1016	590.425	1.29754	
199	benzaldehyde	C100527	C7H6O	106.1	951.8	467.831	1.456	
200	3-ethylpyridine	C536787	C7H9N	107.2	985	530.455	1.511	
201	5-methyl-2-thiophenecarboxal-dehyde	C13679704	C6H6OS	126.2	1099.6	774.426	1.57651	
202	linalool oxide	C60047178	C10H18O2	170.3	1074.7	714.267	1.81291	
203	38	unidentified	-	0	780.4	247.174	1.31617	
204	3-butenenitrile	C109751	C4H5N	67.1	638.2	153.66	1.11763	
205	acetic acid ethyl ester	C141786	C4H8O2	88.1	598.9	139.364	1.10068	
206	ethyl pyruvate	C617356	C5H8O3	116.1	740.1	211.94	1.44238	
207	39	unidentified	-	0	730.7	204.507	1.30099	
202	linalool oxide	C60047178	C10H18O2	170.3	1074.7	714.267	1.81291	
203	38	unidentified	-	0	780.4	247.174	1.31617	
204	3-butenenitrile	C109751	C4H5N	67.1	638.2	153.66	1.11763	
205	acetic acid ethyl ester	C141786	C4H8O2	88.1	598.9	139.364	1.10068	
206	ethyl pyruvate	C617356	C5H8O3	116.1	740.1	211.94	1.44238	
207	39	unidentified	-	0	730.7	204.507	1.30099	
208	3-methyl-3-buten-1-ol	C763326	C5H10O	86.1	733	206.328	1.15737	
209	methyl isobutyl ketone	C108101	C6H12O	100.2	725.6	200.565	1.18372	
210	40	unidentified	-	0	740.5	212.276	1.50986	
211	ethyl 2-methylbutyrate	C7452791	C7H14O2	130.2	847.1	316.211	1.6434	
212	41	unidentified	-	0	846.7	315.8	1.40524	
213	2-furanmethanol	C98000	C5H6O2	98.1	856.6	327.495	1.37841	
214	2,5-dimethylpyrazine	C123320	C6H8N2	108.1	891	371.839	1.49567	
215	heptan-2-ol	C543497	C7H16O	116.2	885.6	364.509	1.37943	
216	1-butanol	C71363	C4H10O	74.1	653.3	159.55	1.37287	
217	42	unidentified	-	0	895.7	378.386	1.58943	
218	β-myrcene	C123353	C10H16	136.2	977.8	516.368	1.28381	
219	pentanoic acid	C109524	C5H10O2	102.1	909.4	398.544	1.2264	
220	2-methylbutanal	C96173	C5H10O	86.1	678.4	169.822	1.16697	

6. Locations of homologues in HS-GC-IMS topographic plots (Figure S2)

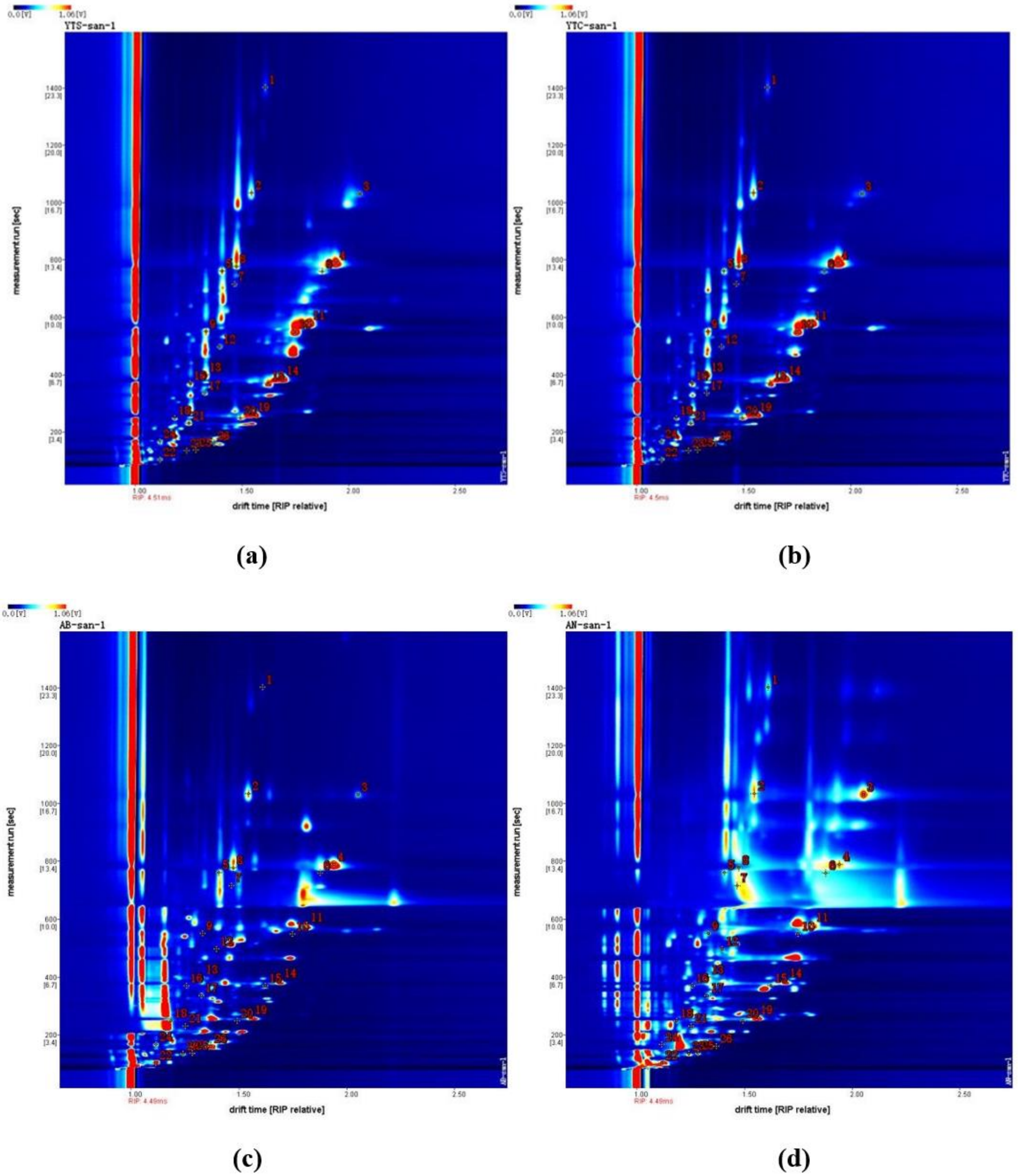

Figure S2. Locations of homologues in HS-GC-IMS topographic plots of different chitosan samples. (**a**) YTS-san; (**b**) YTC-san; (**c**) AB-san; (**d**) AN-san. Locations are marked with cross symbols.

7. Whole gallery plot of chitosan (Figure S3)

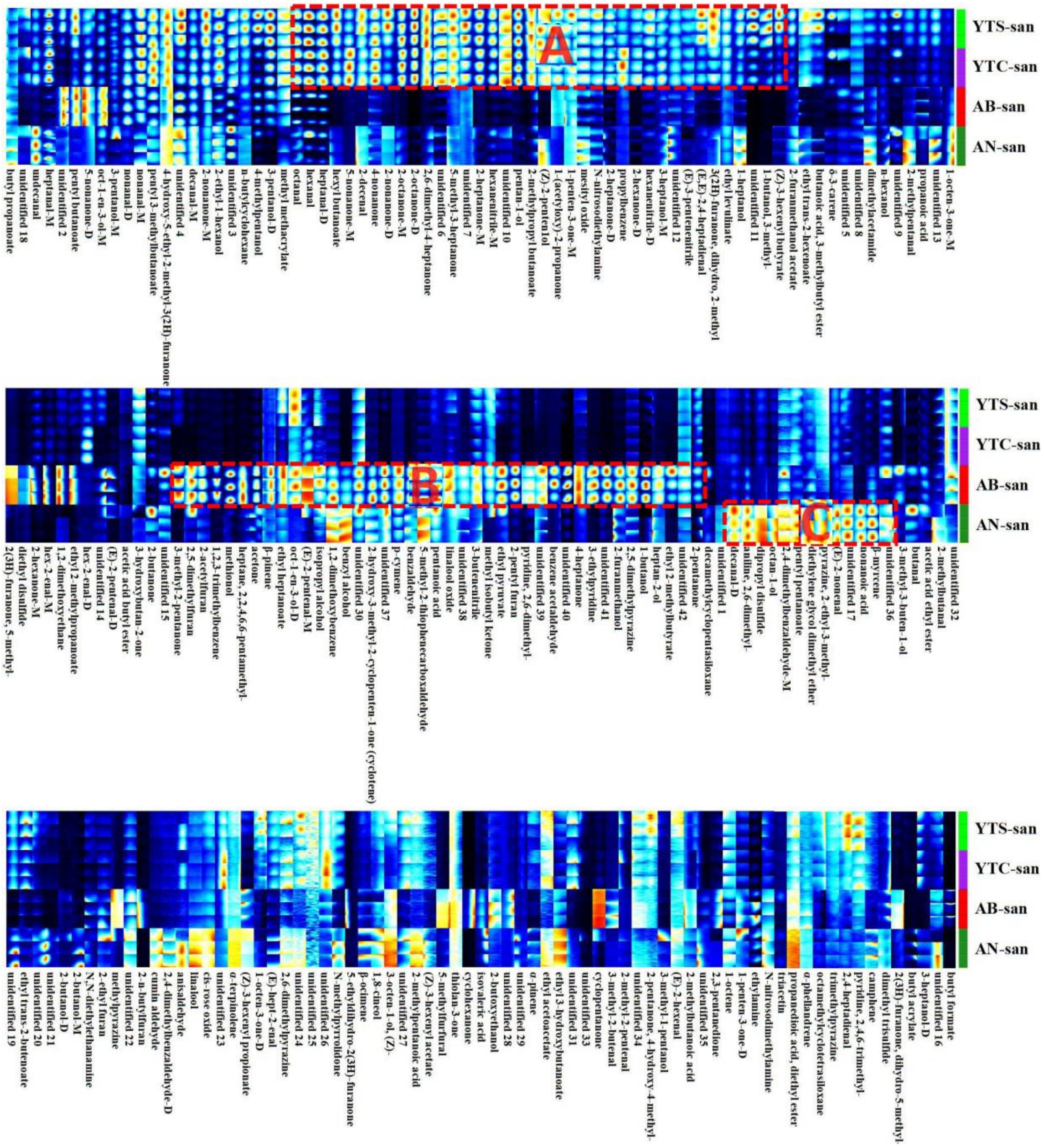

Figure S3. The whole gallery plot of 220 VOCs shows fingerprints of shrimp chitosan (YTS-san), crab chitosan (YTC-san), agaricus bisporus umbrella chitosan (AB-san), and aspergillus niger mycelium chitosan (AN-san). The intensity of the ion signal is reflected by the different colors.

8. Loading scatter plot of the OPLS-DA model (Figure S4, Figure S5)

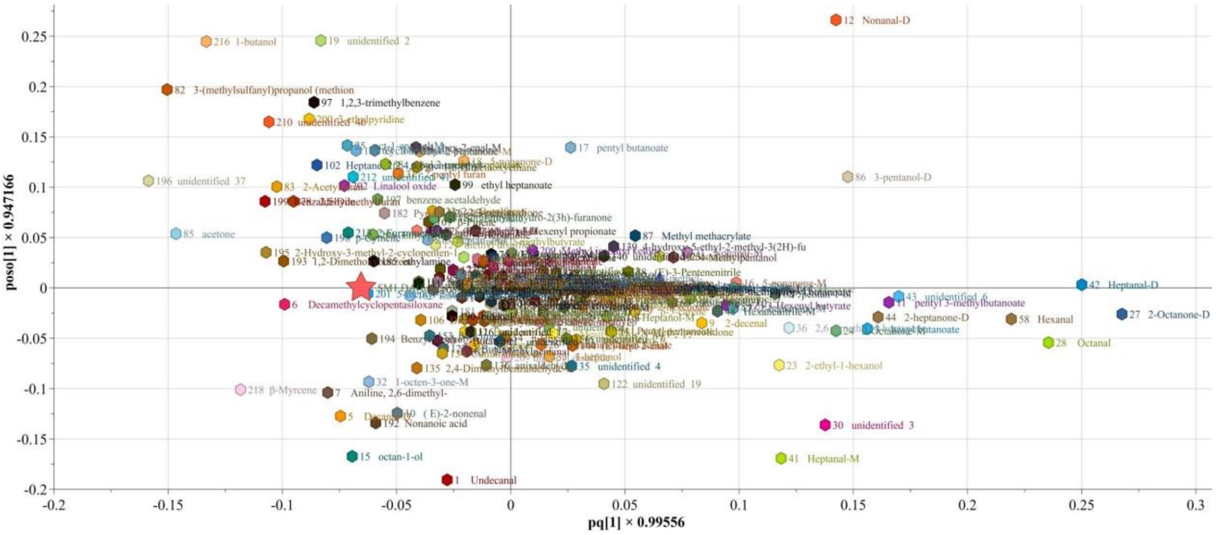

Figure S4. Loading scatter plot of OPLS-DA model. A total of 220 VOCs and groups of chitosan are shown by small hexagons and big stars, respectively.

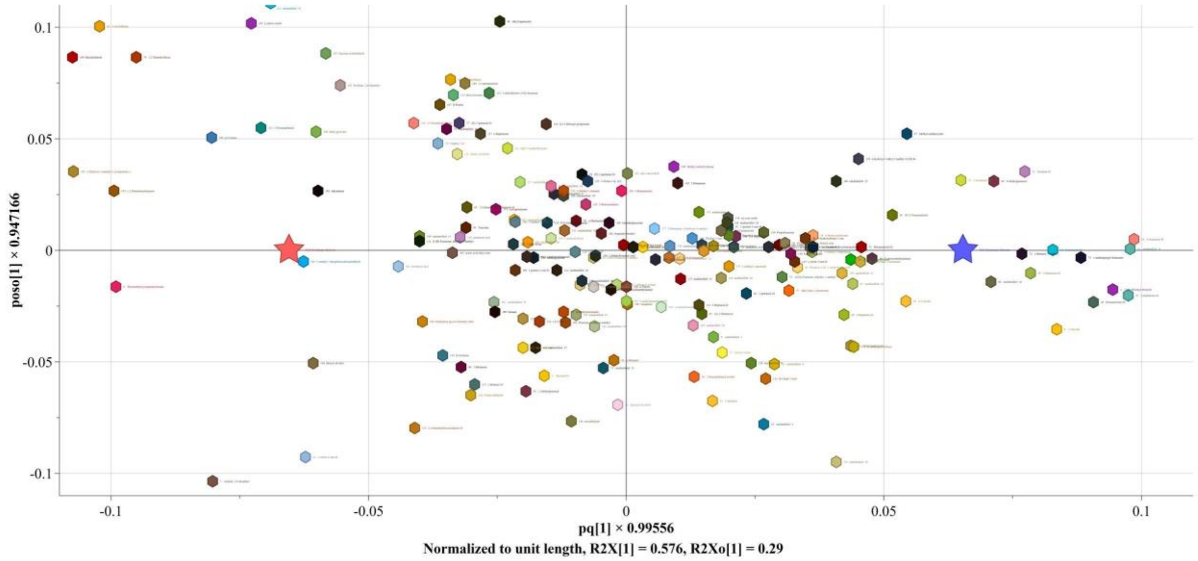

Figure S5. Partial magnification of loading scatter plot of different VOCs. The labels of VOCs are at a smaller point size for clarity.

9. VOCs with high contributions in the OPLS-DA model (Table S3, Figure S6)

NO.	Compound name	VIP	*P*
27	2-octanone-D	3.989160061	3.74103E-09
42	heptanal-D	3.727080107	2.43696E-09
28	octanal	3.508850098	1.17282E-08
58	hexanal	3.267509937	2.51662E-05
43	unidentified 6	2.532320023	4.16034E-10
11	pentyl 3-methylbutanoate	2.466850042	9.91015E-07
44	2-heptanone-D	2.396899939	1.36347E-07
196	unidentified 37	2.36187005	9.79418E-05
3	hexyl butanoate	2.328900099	3.23844E-05
82	3-(methylsulfanyl)propanol (methionol)	2.241559982	0.006549
86	3-pentanol-D	2.197249889	0.000860636
85	acetone	2.186579943	6.78416E-06
24	2-octanone-M	2.122400045	1.72719E-07
30	unidentified 3	2.052279949	0.00630934
216	1-butanol	1.988770008	0.039059602
36	2,6-dimethyl-4-heptanone	1.812319994	1.10996E-06
41	heptanal-M	1.763200045	0.0107735
218	β-Myrcene	1.760519981	0.000436603
23	2-ethyl-1-hexanol	1.752900004	7.28168E-05
22	4-nonanone	1.728119969	9.14608E-08
62	pentan-1-ol	1.724650025	2.50245E-10
199	benzaldehyde	1.600419998	0.000345883
195	2-hydroxy-3-methyl-2-cyclopenten-1-one (cyclotene)	1.597090006	1.30166E-05
210	unidentified 40	1.579949975	0.0170091
83	2-acetylfuran	1.523110032	0.00117748
193	1,2-Dimethoxybenzene	1.480280042	4.95403E-06
6	decamethylcyclopentasiloxane	1.474149942	0.017974701
16	5-nonanone-M	1.466799974	6.99888E-05
61	unidentified 11	1.456609964	0.000189096
47	2-heptanone-M	1.450000048	3.45923E-09
78	2,5-dimethylfuran	1.415699959	0.00174798
4	(Z)-3-Hexenyl butyrate	1.405539989	0.0107014
48	hexanenitrile-M	1.349550009	9.81403E-06
200	3-ethylpyridine	1.316030025	0.0401012
75	2-methylpropyl butanoate	1.315070033	1.73552E-09
102	heptane, 2,2,4,6,6-pentamethyl-	1.26681006	0.0132345
9	2-decenal	1.245159984	4.7741E-05
14	2-nonanone-D	1.23266995	0.000619391
198	p-cymene	1.199710011	4.68333E-05
7	aniline, 2,6-dimethyl-	1.194829941	0.00662352
59	2-hexanone-D	1.167870045	2.65041E-07
21	nonanal-M	1.152109981	5.77913E-05
74	1-butanol, 3-methyl-	1.142950058	0.00204173

Table S3. VOCs of chitosan samples with *P* < 0.05 and VIP > 1.00 in the OPLS-DA model.

5	decanal-D	1.113010049	0.028611099
202	linalool oxide	1.084249973	0.0103756
49	4-methylpentanol	1.06377995	0.045204401
213	2-furanmethanol	1.056900024	0.000191532
60	unidentified 10	1.054530025	3.5686E-07
212	unidentified 41	1.026600003	0.0200324

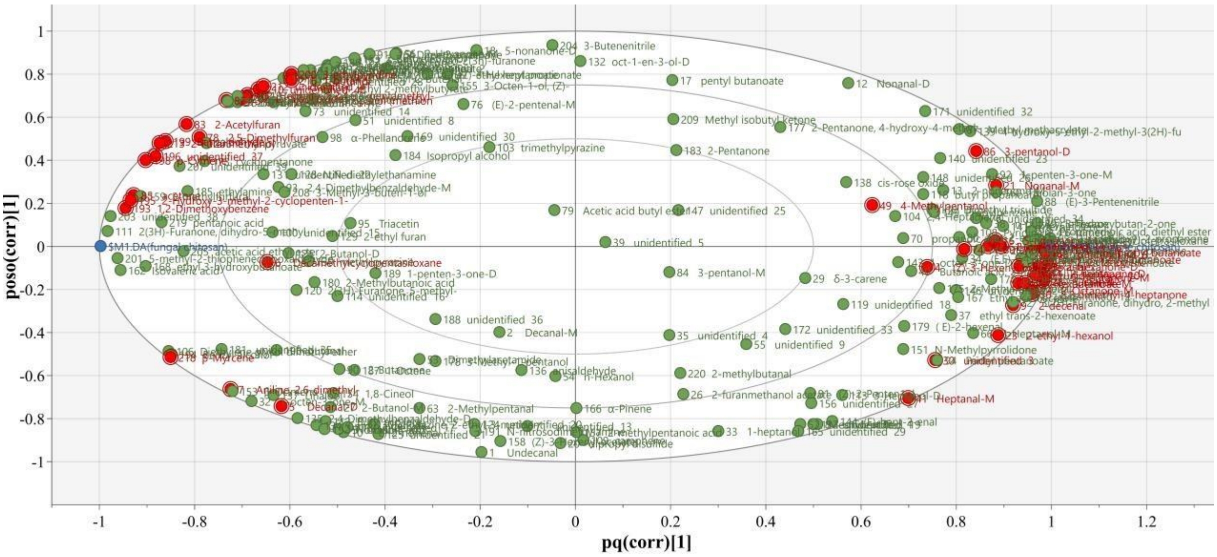

Figure S6. Correlation scaled loadings of the OPLS-DA model, in which red dots represent VOCs with calculation results of *P* < 0.05 and VIP > 1.00.

10. Predicted results of the source identification model (Table S4)

Table S4. Predicted results of work set, test set, and class set.

Samples	YPred (shrimp/crab chitosan)	YPred (fungal chitosan)	Set	Class
YTS-san-1	1.052809954	-0.0528116	work set	shrimp/crab chitosan
YTS-san-2	1.042449951	-0.042450201	work set	shrimp/crab chitosan
YTS-san-3	0.954253972	0.045745902	work set	shrimp/crab chitosan
YTC-san-1	0.975407004	0.024592999	work set	shrimp/crab chitosan
YTC-san-2	0.943363011	0.056636799	work set	shrimp/crab chitosan
YTC-san-3	1.018370032	-0.018367	work set	shrimp/crab chitosan
AB-san-1	0.0212689	0.978730977	work set	fungal chitosan
AB-san-2	-0.00321704	1.003219962	work set	fungal chitosan
AB-san-3	-0.0125719	1.012570024	work set	fungal chitosan
AN-san-1	0.037881698	0.96211803	work set	fungal chitosan
AN-san-2	-0.0168608	1.016860008	work set	fungal chitosan
AN-san-3	-0.013154	1.013149977	work set	fungal chitosan
CTS1-1	0.668880999	0.331119001	test set	shrimp/crab chitosan
CTS1-2	0.740056992	0.259943008	test set	shrimp/crab chitosan
CTS1-3	0.715327978	0.284671992	test set	shrimp/crab chitosan
CTS2-1	0.663751006	0.336248994	test set	shrimp/crab chitosan
CTS2-2	0.728628993	0.271371007	test set	shrimp/crab chitosan
CTS2-3	0.720606029	0.279394001	test set	shrimp/crab chitosan
CTS3-1	0.770485997	0.229514003	test set	shrimp/crab chitosan

CTS3-2	0.750937998	0.249062002	test set	shrimp/crab chitosan
CTS3-3	0.695523977	0.304475993	test set	shrimp/crab chitosan
CTS4-1	0.889710009	0.110289998	test set	shrimp/crab chitosan
CTS4-2	0.825501978	0.174498007	test set	shrimp/crab chitosan
CTS4-3	0.836980999	0.163019001	test set	shrimp/crab chitosan
MF-1	0.178867996	0.821132004	class set	fungal chitosan
MF-2	0.260497004	0.739503026	class set	fungal chitosan
MF-3	0.269488007	0.730512023	class set	fungal chitosan
FQ-1	0.934534013	0.0654663	class set	shrimp/crab chitosan
FQ-2	0.843611002	0.156389996	class set	shrimp/crab chitosan
FQ-3	0.883009017	0.116990998	class set	shrimp/crab chitosan
PBM-1	0.764235973	0.235763997	class set	shrimp/crab chitosan
PBM-2	0.905228019	0.0947721	class set	shrimp/crab chitosan
PBM-3	0.887171984	0.112828001	class set	shrimp/crab chitosan

Disclaimer/Publisher's Note: The statements, opinions and data contained in all publications are solely those of the individual author(s) and contributor(s) and not of MDPI and/or the editor(s). MDPI and/or the editor(s) disclaim responsibility for any injury to people or property resulting from any ideas, methods, instructions or products referred to in the content.
